# Analysis of a Community Health Screening Program and the Factors Affecting Access to Care

**DOI:** 10.7759/cureus.41907

**Published:** 2023-07-14

**Authors:** Connor D Fritz, Jeanana Khan, Panayiotis D Kontoyiannis, Emily M Cao, Alexandria Lawrence, LaTanya D Love

**Affiliations:** 1 Public Health, University of Texas Health Science Center at Houston John P. and Katherine G. McGovern Medical School, Houston, USA; 2 Dermatology, University of Texas Health Science Center at Houston John P. and Katherine G. McGovern Medical School, Houston, USA; 3 Pediatrics, University of Texas Health Science Center at Houston John P. and Katherine G. McGovern Medical School, Houston, USA

**Keywords:** public health, family medicine, health fair, preventative medicine, community health, health disparities, primary care

## Abstract

Introduction

Free community health fairs and screening initiatives can be effective in broadening access to care and improving health outcomes in historically marginalized communities. UTHealthCares is a community health-focused organization developed at the University of Texas Health Science Center in Houston. At the beginning of 2023, UTHealthCares oversaw a free community health fair in the Eastex-Jensen Area - a medically underserved area in Northeast Houston. The health fair consisted of four stations - vitals and body mass index collection, vision screening, blood glucose screening, and dental screening. Participants also received coronavirus disease 2019 vaccinations, referrals, and health education. The purpose of this study is to evaluate the effectiveness of the UTHealthCares community health fair while assessing the factors that influence participants’ access to medical care.

Methods

After completing the health fair, participants filled out an optional questionnaire. The questionnaire contained items that assessed satisfaction with the health fair, improvements in managing health, and access to resources. We calculated descriptive statistics, including mean response and 95% confidence intervals for rating scale questions. We used the chi-squared test to evaluate the independence of categorical variables and the Mann-Whitney U test to evaluate differences in means between distributions.

Results

A total of 111 people participated in the health fair, 91 of which completed a questionnaire. When participants rated their satisfaction with the health fair, the average response was 4.62 out of five. Participants also reported that they were more comfortable managing areas of health related to the stations offered at the fair. Many participants reported limited access to fresh food and long travel times to the physician. Participants that traveled further to reach one resource also tended to have significantly higher travel times for the other: X2 (4, N=78)=28.04, p<0.0001. However, 77.8% of respondents reported that the lack of insurance or cost was their greatest barrier to seeing a medical provider, while only 2.47% reported the lack of transportation as their greatest barrier. Participants who reported having health insurance also had a significantly higher probability of visiting a medical provider when they had a health issue: U=928.5, p=0.0006.

Conclusion

Overall, participants reported high satisfaction with the health fair. Participants also gave valuable feedback for improving future community health initiatives. Although many participants reported travel times greater than 30 minutes to reach community resources, very few participants indicated that transportation was their largest barrier to accessing medical care. Instead, the lack of insurance and high costs seem to be participants’ most significant hindrances. Therefore, interventions in the Eastex-Jensen area focused on expanding access to care should also include components that improve access to insurance.

## Introduction

Community health fairs - short-term events in which participants receive free medical services, education, and community resources - can be an effective way to provide healthcare resources to people from underserved or historically marginalized communities. Attendees of health fairs report following up with physician referrals, enrolling in health insurance, and making lifestyle improvements based on the information provided at health fairs [1]. Furthermore, information collected from health fair participants can provide insight into community member demographics, health outcomes, and social determinants of health [1,2]. This information, in turn, can improve further community-based initiatives by inducing the proper allocation of resources commonly lacking by people in these communities.

Providing health education, such as nutritional counseling or knowledge of disease prevention, is often an important component of community health fairs. Education provided through health fairs improves participants’ health knowledge in domains such as cardiovascular disease, contraception, dental health, diabetes, substance abuse, and nutrition [3]. Because limited health literacy is a driver of poor health outcomes, such as heart failure [4], periodontal disease [5], and poor glycemic control in diabetes [6], initiatives that aim to improve health literacy in underserved populations may be an effective way of reducing the health inequities faced by people within these populations.

Health fairs often provide free routine medical services and preventative screening to participants. These services may include blood glucose checks, mammograms, eye exams, or cholesterol checks. As many health fair participants report never - or rarely - receiving preventative health care check-ups, health fairs can provide preventative care for people who would not otherwise receive it [7]. Health fairs have also been shown to be an effective method for broadening access to cancer screening in medically underserved communities [8,9]. As earlier cancer detection is associated with lower morbidity and mortality, providing free cancer screening is directly beneficial to the health of the communities where these services are offered. Many health fairs also offer participants free vaccinations, and larger curbside/drive-through vaccination clinics have increased in frequency in recent years due to the coronavirus disease 2019 (COVID-19) pandemic. Such events increase vaccination rates in local populations, and some organizations have used these events to provide medical care and health education, in addition to vaccinations, to great effect [10].

Some health fairs may be staffed by health professions students. That is, many of the volunteers providing the services and education at these events are students training to become physicians, nurses, dentists, or other healthcare providers. While providing a direct benefit to the community in which they are held, these types of health fairs also provide substantial benefits to the student volunteers who participate. However, it is important that these students have direct supervision from more experienced clinicians to prevent errors in testing or education. When done appropriately, student involvement in community health fairs allows health professions students to gain clinical experience under the supervision of experienced healthcare providers [3]. Health fairs may also provide students with the opportunity to interact with members from communities they may not otherwise interact with in their clinical coursework. For instance, students involved in a health fair in a US-Mexico border community reported viewing their participation as a valuable educational experience. These students specifically felt they improved their awareness of community-based resources available for underserved people because of their participation [11]. Student involvement in health fairs and other community-focused health initiatives can also increase student interest in community health [12]. This effect could potentially lead to a higher number of healthcare providers that are competent in providing care to underserved individuals.

UTHealthCares and the Eastex-Jensen area

Racial and ethnic minorities [13], people with limited English proficiency [14], and people with low income [15] face substantial barriers to accessing healthcare in the United States. Providing free health services through initiatives such as health fairs can partially address some of the inequities faced by these populations by expanding access to screening services, providing referrals, and improving health education.

Started in 2018, UTHealthCares is an interprofessional organization run by students and faculty from professional schools under the University of Texas Health Science Center at Houston (UTHealth) umbrella. The focus of UTHealthCares is to organize an annual health fair in a medically underserved area near Houston. In 2021 and 2022, the health fair was put on hiatus due to social distancing measures caused by the COVID-19 pandemic. However, UTHealthCares was renewed at the end of 2022 and hosted the next iteration of the annual health fair in February 2023 [16].

The 2023 UTHealthCares health fair was held at the UTPhysicians Multispecialty Clinic in the Eastex-Jensen area. The Eastex-Jensen area is a super neighborhood - or neighborhood planning area - in Northeast Houston. The Eastex-Jensen area exists within a medically underserved area (MUA), a dental health professional shortage area (HPSA), and a mental health HPSA. These classifications, made by the Health Resources and Service Administration, mean that people who live in the Eastex-Jensen area have limited access to primary care, dental care, and mental health services [16]. Households within the Eastex-Jensen community also tend to have lower incomes than households in the broader Houston area. As of 2019, the median household income in the Eastex-Jensen area was $38,044 per year, compared to Houston’s median household income of $52,338 per year. Additionally, 39% of households in the Eastex-Jensen community have an annual household income under $25,000, compared to 24% of households in Houston as a whole [17]. The racial and ethnic demographics of the Eastex-Jensen area and Houston also differ. In the Eastex-Jensen area, Hispanic people make up 76% of the population, non-Hispanic Black people make up 18% of the population, and non-Hispanic White people make up 6% of the population. In Houston, Hispanic people make up 37% of the population, non-Hispanic Black people make up 25% of the population, and non-Hispanic White people make up 31% of the population. In the Eastex-Jensen Area, 68% of households speak Spanish as their primary language, 31% speak English, and 1% speak another language [17].

The current study evaluated the 2023 UTHealthCares community health fair by assessing a questionnaire administered after participants completed the fair. Using the questionnaire results, we examined participant satisfaction, participant recommendations for future events, and the barriers that affect participants’ health and access to care. We found that participants were highly satisfied and offered valuable insights for improving future community health events. While many participants had long travel times to access fresh food and medical care, their main barrier to accessing care was the lack of insurance or high cost.

## Materials and methods

Participants were recruited through paper fliers and local television advertisements. Paper fliers were distributed at the UTPhysicians Multispecialty Clinic and at schools and churches in the Eastex-Jensen area. There were no specific exclusion or inclusion criteria for participation in the health fair. Participants in the health fair went through four stations: vitals and body mass index (BMI) collection, vision screening, blood glucose testing, and dental screening. Descriptions of each of the stations are shown below in Table 1. Each station was operated by two or more volunteers, at least one of which was fluent in Spanish. Volunteers were either medical or dental students, depending on their assigned station. Physicians and dentists were also present to oversee students and provide care in emergencies.

**Table 1:** Descriptions of the services offered at each of the four health fair stations.

Station	Components
Vitals and BMI	1. Height and weight collection with BMI calculation
	2. Oral temperature, blood pressure, respiratory rate, and heart rate collection
	3. Education on normal ranges of vital signs
Vision Screening	1. Visual acuity and visual field screening
	2. Cranial nerve II, III, IV, and VI screening
	3. Eye pressure screening with TonoPen
	4. Education about the results and visual health
Blood Glucose	1. Blood glucose measurement
	2. Education about preventing and managing diabetes
Dental Screening	1. Dental exam
	2. Fluoride treatment
	3. Provide resources about dental hygiene, local community clinics, and low-cost options for care

To improve participants' throughput during the health fair, organizers created three parallel streams of the four stations. At check-in, volunteers assigned each participant to one of the three streams. After completing their stations, participants aggregated in a larger room where volunteers gave them educational materials and general counseling related to dental hygiene, local courses at a community college, nutrition, and exercise. Participants were also offered free COVID-19 vaccinations and follow-up appointments at the UTPhysicians Multispecialty Clinic. A separate room was allocated for patients with emergency health concerns. Additionally, UTHealthCares established a “Teddy Bear Clinic” for pediatric participants at the health fair. Volunteers calculated BMI, collected vital signs (oral temperature, blood pressure, heart rate, and respiratory rate), and handed out stuffed teddy bears to each of the children whose parents wished to participate in the clinic. In 2023, the UTHealthCares health fair had 111 adult participants.

After participants finished receiving educational materials at the last station of the health fair, they were asked to complete optional paper questionnaires. Both English and Spanish versions were offered to participants. The questionnaires did not solicit any information that could be used to identify health fair participants. Instead, they consisted of a series of rating scales, free response, and interval scale questions that assessed participants' attitudes towards the health fair and their perceived access to community resources.

Questionnaire creation

The questionnaires were created through an iterative process with feedback from community practice managers and community health educators affiliated with the UTPhysicians Multispecialty Clinic. These individuals had extensive experience organizing events in the Eastex-Jensen community and had been key contributors to organizing the previous UTHealthCares health fairs. The items comprising the initial questionnaire were sent to these contributors, who would then offer changes, suggestions to remove questions, or suggestions to add questions. After these changes had been addressed, the new version was sent out to each contributor. This process continued until the contributors had no more suggestions. Overall, suggestions primarily focused on making the questionnaire more understandable and removing items related to factors not addressed by the health fair, such as participants’ changes to healthcare access due to COVID-19. The result of this process was an English questionnaire. This English version was then translated to Spanish using Google Translate. Each item was translated individually until a preliminary Spanish version was completed. The English questionnaire and the preliminary Spanish version were sent to two independent native Spanish speakers who were not otherwise affiliated with UTHealthCares. These individuals each provided corrections to the initial translated questionnaire, which were incorporated into the final Spanish version that was distributed at the health fair.

Questionnaire contents

The final questionnaire consisted of 13 items that assessed participants’ satisfaction with the health fair, recommendations for the health fair, and access to health-related resources. Questions were either rating scale questions, interval scale questions, free response questions, or yes/no questions. For rating scale questions, participants chose a single response to a question from a set list of options. For example, participants could choose from the options “Never,” “Rarely,” “Likely,” and “Always” when asked the question “How likely are you to go to the doctor if you develop a medical issue?” Interval scale questions involved participants choosing a number to represent their response to a given question. As an example, participants could choose a number from 0 to 10 to respond to the question: “What is your family’s access to fresh food, on a scale from 0 to 10?” Free response questions involve participants writing text in response to a question. The 13 questions included in the questionnaire are listed in Table 2 below.

**Table 2:** Items comprising the questionnaire offered to health fair participants.

Item	Answer Choices
1. How satisfied are you with the health fair today?	Very Dissatisfied | Dissatisfied | Neither Satisfied nor Dissatisfied | Satisfied | Very Satisfied
2. For each booth, how much more comfortable are you in managing these areas:	
Vitals & BMI?	Not Comfortable | Less Comfortable | No Change | More Comfortable | Very Comfortable
Dental Care?	Not Comfortable | Less Comfortable | No Change | More Comfortable | Very Comfortable
Vision Care?	Not Comfortable | Less Comfortable | No Change | More Comfortable | Very Comfortable
Blood Glucose?	Not Comfortable | Less Comfortable | No Change | More Comfortable | Very Comfortable
Accessing Health Info?	Not Comfortable | Less Comfortable | No Change | More Comfortable | Very Comfortable
3. Is there anything specific that you had hoped to learn/see that we did not include?	(Free Response)
4. Any health services you would like more access to?	(Free Response)
5. What is your family's access to fresh food, on a scale from 0 to 10?	A scale from 0 to 10, with 0 corresponding to “No change in access” and 10 corresponding to “Much harder to access”
6. How far do you have to travel for fresh produce?	Under 10 minutes | 10-30 minutes | Over 30 minutes
7. How many meals do you use fresh produce for instead of canned goods, frozen foods (excluding fruits and vegetables), etc.?	None | Very few | Some meals | Most meals | All meals
8. How far do you have to travel to see the doctor?	Under 10 minutes | 10-30 minutes | Over 30 minutes
9. How likely are you to go to the doctor if you develop a medical issue?	Never | Rarely | Likely | Always
10. What is your biggest barrier to seeing a doctor? (pick one)	Hours of operation/Work schedule | Cost/Insurance | Transportation | Obligations at home | Other: _____________
11. Do you have health insurance?	Yes | No
12. If no, what are the barriers to your access?	(Free Response)
13. If yes, are the providers near you in-network?	Yes | No

Analysis

After the volunteers collected the completed questionnaires at the health fair, we transcribed each one from a paper format to an electronic format using Qualtrics [18]. We kept the free-response questions in their original form when entering them into Qualtrics. However, we translated Spanish responses to English when categorizing and counting question responses. After we transcribed each questionnaire, we downloaded the responses as a comma-separated values file and performed statistical analysis using Python 3.9.

To analyze participant responses to the rating scale questions, we converted each response option to a number from one to five, with more negative responses corresponding to smaller numbers. For example, the responses to question nine were “Never,” “Rarely,” “Likely,” and “Always.” These responses would be converted to numbers one, two, three, and four, respectively. After converting each response, we calculated the mean response for each rating scale and interval scale question. For the responses to these types of questions, we also calculated the 95% confidence interval of the mean response using a normal distribution. For certain rating scale questions, we also calculated the proportion of participants that chose each response.

When analyzing the free-response questions, we subjectively categorized each response based on the main topic mentioned in the response. For example, we categorized the responses “chequeo de cholesterol” and “examen de cholesterol” as “cholesterol.” We discarded non-specific responses and responses that did not answer the question. As an example, when categorizing the responses to question three, we ignored the responses “no,” “all is good,” and “más serbicios.” We then counted the number of responses that qualified for the categories related to each question.

We used the chi-squared test [19] to test for independence between categorical variables that each had more than two groups. When comparing two categorical variables, one of which had two groups, we used the Mann-Whitney U test [20] to test if the means of the distributions of the groups differed significantly.

## Results

After collecting and transcribing the questionnaires, we had 91 responses from 111 total health fair participants. We measured the overall participant satisfaction with the health fair and satisfaction with the individual stations using questionnaire items one and two. The mean response to each of these items is reported in Table 3, along with a 95% confidence interval for each mean. Participants reported high satisfaction, with a mean response of 4.62 out of five, with five corresponding to the response “very satisfied.” On average, participants reported feeling much more comfortable managing the areas of health covered by the health fair stations they participated in. The mean responses to the questions that assessed comfort in managing health-related areas ranged from 4.82 to 4.87 out of five, with five corresponding to the response “very comfortable.”

**Table 3:** Average participant responses to questionnaire items one and two.

Item	Mean	95% CI
1. How satisfied are you with the health fair today?	4.62 (N=85)	(4.40, 4.85)
2. For each booth, how much more comfortable are you in managing these areas:		
Vitals & BMI?	4.87 (N=89)	(4.79, 4.94)
Dental Care?	4.83 (N=89)	(4.72, 4.94)
Vision Care?	4.82 (N=88)	(4.70, 4.93)
Blood Glucose?	4.85 (N=89)	(4.74, 4.96)
Accessing Health Info?	4.89 (N=89)	(4.81, 4.96)

**Figure 1:**
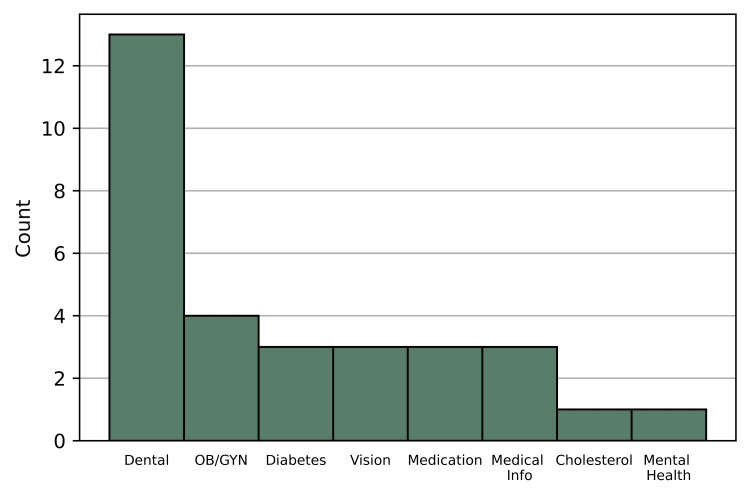
Counts of response type to questionnaire item 4: “Any health services you would like more access to?" (N=31).

Although participants reported high satisfaction with the health fair, nine participants also offered suggestions for improvement when prompted through question three: “Is there anything specific that you had hoped to learn/see that we did not include?.” The most common suggestion - with three respondents - was to include cholesterol checks. Other suggestions for future inclusions were toenail treatment, obstetrics and gynecology (OB-GYN) services, behavioral health services, dermatology services, flu shots, and cardiology services. Each of these was suggested by a single respondent. Many of the participants also reported healthcare services they would like access to more generally. These 31 responses are summarized in Figure 1. The most commonly mentioned services were related to dental care - these made up nearly 42% of the responses. However, participants expressed a desire for access to a broad range of services. Some participants mentioned wanting access to services related to OB-GYN, such as mammograms. Other participants wanted better access to blood sugar checks to control their diabetes, medical and visual prescriptions, and medical information. Only one person mentioned wanting better access to cholesterol checks, despite this being the most commonly requested improvement to the health fair.

**Figure 2:**
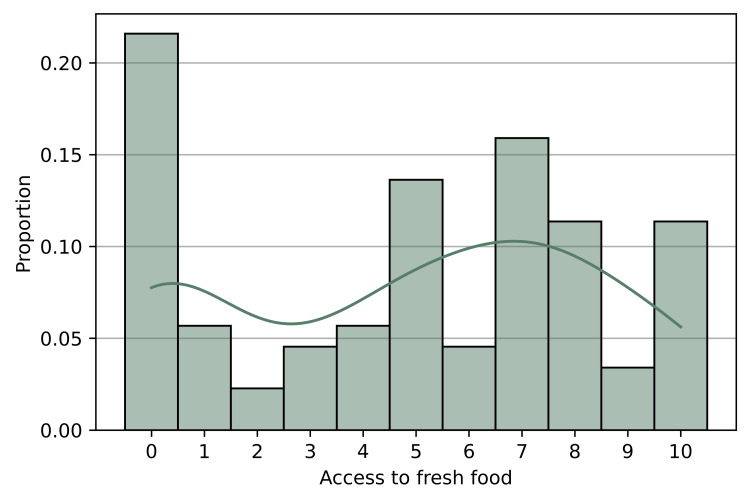
Participants' responses to questionnaire item five: “What is your family's access to fresh food, on a scale from 0 to 10?” (N=88). The continuous line indicates the kernel density function of the proportion of participant responses. The kernel density function is used to smooth the distribution to more clearly show the bimodal nature of participants' responses.

When asked to rate their access to fresh food through question five, 88 participants chose values that created a bimodal distribution of the responses shown in Figure 2. While many participants rated their access to fresh food around a seven out of 10, the average for all responses was 4.88. This is because 27.3% of respondents chose either a zero or a one to characterize their access to fresh food. Many participants also indicated that they experienced long travel times if they wanted to buy fresh produce. Moreover, 12.2% of respondents said they had to travel over 30 minutes, 43.3% said they had to travel 10-30 minutes, and 44.4% said they could buy fresh food after traveling less than 10 minutes. Additionally, 17.9% of respondents reported that they had no, or very few, meals that utilized fresh produce.

**Figure 3:**
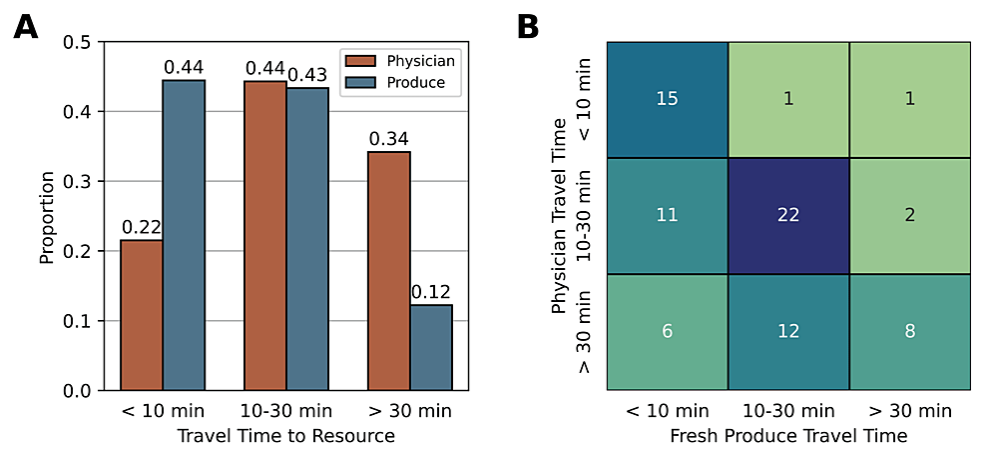
Comparisons of travel times to different resources. (A) A comparison of participants' travel time to the physician and their travel time to access fresh produce. Specifically, 34.2% of participants travel over 30 minutes to reach a physician, and 12.2% of respondents travel over 30 minutes to reach fresh produce. (B) A cross-tabulation of travel times to each resource. There is a significant positive association between travel times. X2 (4, N=78)=28.04, p<0.0001

Many respondents also reported long travel times to see a physician. When answering question eight, 79 participants responded, and 34.2% of respondents reported that they had to travel over 30 minutes to see a physician, 44.3% reported that they had to travel 10-30 minutes, and 21.5% reported they had to travel less than 10 minutes. These results are shown in Figure 3A and are compared to the fresh produce travel times also reported by participants. Although more participants reported higher travel times to reach a physician, those participants travel further to reach one resource also tended to have significantly higher travel times for the other, X2 (4, N=78) = 28.04, p<0.0001. For example, 72% of respondents that reported traveling over 30 minutes to reach fresh produce also reported traveling over 30 minutes to see a physician. However, only 18.8% of respondents who reported traveling under 10 minutes to find fresh produce reported a physician travel time of over 30 minutes. A cross-tabulation of travel times demonstrating this pattern is shown in Figure 3B.

When answering question nine, 22.3% of respondents reported that they never, or rarely, visited a physician if they developed a medical problem. Although there is a significant relationship between travel time to reach fresh produce, and travel time to reach a physician, we did not find a significant relationship between travel time to reach a physician and respondents’ self-rated likelihood of visiting a physician if they had a medical problem: X2 (6, N=78) = 4.55, p=0.602. Although this result is somewhat counterintuitive, when responding to question 10, 77.8% of respondents reported that the lack of insurance or cost was their largest barrier to seeing a medical provider. Only two out of 81 respondents reported transportation as their largest barrier to seeing a physician. In response to question 11, 77% of respondents reported that they did not have insurance. Of the 67 participants who reported that they did not have insurance, 42 explained their barriers to insurance access in question 12. The most common barrier had to do with cost - 35 respondents reported that they could not access insurance because of a lack of employment or a lack of money. Meanwhile, five respondents reported that they could not access insurance due to their immigration status, and two respondents reported that they did not know how to access insurance. Additionally, 48.3% of respondents reported that they did not have a nearby, in-network provider. Participants who reported having health insurance also had a significantly higher self-rated likelihood of visiting the physician when they had a medical issue than participants without insurance: U=928.5, p=0.0006. Participants who reported having health insurance rated their likelihood of visiting a healthcare provider if they had a medical problem as 3.5 out of four, on average. Participants who did not have health insurance rated the same likelihood as 2.79 out of four, on average.

## Discussion

Overall, participants reported high general satisfaction with the health fair. Participants also reported they were more comfortable managing areas of health related to the stations offered at the fair. These results suggest that health fairs held in the Eastex-Jensen community may be an effective method for improving participants' knowledge of their own health, which could partially reduce disparities in health outcomes. These findings are in line with the results of other health fairs, which found that participants had improved health knowledge and followed up on provided resources [1,3]. However, participants also indicated improvements that can be made to future student health fairs and the health education offered therein. Many respondents reported that they would have liked the health fair to include cholesterol checks. As these seem to be valuable to patients of this community, it would be beneficial to add cholesterol screening to the next health fair. Indeed, regular cholesterol screening is important for maintaining cardiovascular health [21]. Participants also requested a variety of other services, such as toenail treatments, mammograms, and flu shots - all of which could potentially be included in future community initiatives. Similarly, participants responded with a wide range of answers when asked which health services they would generally like more access to. Although the provided stations related to many of the commonly reported services - such as dental care and diabetes care - the screening services offered at the health fair could not fully meet the needs of participants. For instance, the blood sugar checks offered at the health fair would not fully address the needs of a participant that could not find adequate diabetes care elsewhere. The health education and referrals offered at the fair may close this gap somewhat, but do not fully address many of the barriers that reduce access to care, such as the lack of insurance. Intrinsic qualities of student-run health fairs, however, may present limit the services that can be offered. For example, one suggestion seen in the questionnaires was additional behavioral/mental health services. The provision of in-depth services such as mental and behavioral health treatment may encounter time limitations when it comes to annual student-run health fairs, which are often organized to be short-term, high-throughput events. Addressing mental health issues may take longer than a one-day health fair as therapy can necessitate multiple sessions, and psychiatric medications take longer periods to take effect. Participant responses uncovered potential areas of improvement, especially regarding additional services that could be offered. For future health fairs, organizers should continue surveying the needs of the community to improve services better catered to the local population.

Many respondents reported low access to fresh food, and few meals containing fresh produce. Specifically, 12.2% of respondents reported that they had to travel for greater than 30 minutes to access fresh produce. Therefore, a key issue to address in the Eastex-Jensen community is a lack of access to fresh food and produce. Indeed, food insecurity can lead to, or exacerbate, chronic diseases and conditions, eventually leading to increased avoidable medical expenses [22]. To address this, it may be beneficial to offer education related to accessing fresh food, or referrals to nutrition-focused community resources at future initiatives within the Eastex-Jensen community. Many participants also reported high travel times to their healthcare provider, and there was a significant positive relationship between travel time to buy fresh produce and travel time to a physician. This association may be because some participants live in more isolated areas that are further away from health-related resources in general. Those who live in rural areas [23] and areas further away from healthcare services [24] tend to have worse health outcomes. However, we did not find a significant relationship between respondents’ travel time to reach a physician and their likelihood of visiting a physician if they develop a medical problem. This could be because participants’ barriers to accessing medical care are largely not due to a lack of transportation. Indeed, most respondents reported that the lack of insurance or cost was their largest barrier to seeing a medical provider - only 2.47% of respondents cited a lack of transportation as their largest barrier. These results suggest that community initiatives focused on providing transport may not be an effective method for broadening access to care, at least in the Eastex-Jensen community. Participants who reported having insurance also reported a significantly higher likelihood of visiting a medical provider when they had a medical problem when compared to those participants who did not have access to insurance. Therefore, improving access to insurance may also improve access to care and health-related outcomes in the Eastex-Jensen community. As most respondents reported that cost was their primary obstacle to accessing insurance, one way to potentially improve access would be to provide education about low-cost options for accessing care. For instance, the Harris Health System (HHS) is a healthcare system in Harris County (which includes the Eastex-Jensen area) that offers a financial assistance program on a sliding scale [25]. Initiatives such as the HHS financial assistance program offer enhanced access to care for uninsured or underinsured individuals. Therefore, it is important for providers to be aware of similar programs within their communities. Providing information about these programs should also be an integral part of short-term health-focused initiatives such as community health fairs.

Limitations of the questionnaire itself include a risk of selection bias. Since we particularly targeted the Eastex-Jensen community when advertising the health fair through advertisements on local television and in establishments within the Eastex-Jensen area, the population selected to complete the questionnaire was not random. Therefore, participant responses may not be generalizable to other populations. Administration of the questionnaire also risks self-report bias. As respondents answered the questionnaire items themselves, results may be biased by the patient’s feelings or behaviors at the time of completion. Still, participant responses offer valuable insights into the people who live in the Eastex-Jensen community and attended the health fair. Additionally, responses revealed areas for improvement in future community-based initiatives. Because data were only collected after participants had completed the health fair, future work could use more robust methods to evaluate health fair outcomes - such as the administration of pre- and post-fair surveys. Improved data collection methods would improve the validity of the results observed in this study and provide a stronger case for informing policy or organizational decisions. Future work may also focus on verifying that the results observed in this study generalize to other communities. This could be accomplished by collecting data related to health fairs held in other geographic locations. Understanding how factors such as insurance status influence access to care in diverse communities is important for creating organizations and initiatives to address healthcare disparities. 

## Conclusions

UTHealthCares is an interprofessional organization consisting of students and faculty from professional schools under the University of Texas Health Science Center at Houston umbrella. In February 2023, the members of UTHealthCares organized a single-day community health fair in the Eastex-Jensen area in Northeast Houston. The Eastex-Jensen area exists within an MUA, a dental HPSA, and a mental health HPSA. Additionally, many people living in the Eastex-Jensen area have limited English proficiency, which may limit their access to healthcare. The community health fair consisted of four primary stations - vitals/BMI collection, vision screening, blood glucose screening, and dental screening. Participants were also offered free COVID-19 vaccinations, referrals to a local primary care clinic, and health-related education. Each of the stations was operated by two or more student volunteers, at least one of which was fluent in Spanish. Volunteers were either medical or dental students, depending on their assigned station. Physicians and dentists were also available to oversee volunteers and to provide care in more emergent situations. After completing the health fair, participants were offered questionnaires in either English or Spanish. A total of 111 people participated in the health fair, 91 of which completed a questionnaire. The questionnaire itself consisted of 13 items that assessed respondents’ satisfaction with the health fair, their perceived improvement in managing their health, and their access to specific health-related resources.

The focus of the present study was to evaluate the effectiveness of the health fair and to explore potential improvements for future community health initiatives. We found that participants were overall very satisfied with their experience at the health fair - on average they rated their satisfaction as a 4.62 out of 5. On average, participants also reported that they were more comfortable managing the areas of health covered by the stations offered at the fair. When asked to report on potential improvements to the health fair, participants offered many relevant suggestions for future community health initiatives. These include lipid screening, mammograms, and flu shots. These results demonstrate the value of using participant feedback to inform community initiatives. A second focus of this study was to examine the barriers that affect health and access to care for members of the Eastex-Jensen community. Many participants reported limited access to fresh food and long travel times to the physician. Those participants that had to travel further to reach one resource also tended to have significantly higher travel times for the other: X2 (4, N=78)=28.04, p<0.0001. However, we did not find a significant relationship between respondents’ travel time to reach a physician and their probability of visiting a physician if they had a medical problem: X2 (6, N=78)=4.55, p=0.602. This is likely because the participants’ largest barrier to accessing care was not transportation, but the lack of insurance and cost of care. To support this - 77.8% of respondents reported that the lack of insurance or cost was their largest barrier to seeing a medical provider, while only 2.47% of respondents reported a lack of transportation as their largest barrier. Additionally, participants who reported having health insurance also had a significantly higher probability of visiting a medical provider when they had a health issue than participants without insurance: U=928.5, p=0.0006. As the largest barrier to care for participants was the lack of insurance and high cost, it is important to address this issue when attempting to improve access to care. One potential avenue to improve access in the context of health fairs is to provide education about financial assistance programs and low-cost options for care.
